# Older Black Americans and Depressive Symptoms During the COVID-19 Pandemic

**DOI:** 10.1093/geroni/igab046.3717

**Published:** 2021-12-17

**Authors:** David Chae, Connor Martz, Kara Chung, Roland Thorpe, Karen Lincoln

**Affiliations:** 1 Tulane Universtiy, Tulane University, Louisiana, United States; 2 Tulane University, New Orleans, Louisiana, United States; 3 Johns Hopkins Bloomberg School of Public Health, Johns Hopkins Bloomberg School of Public Health, Maryland, United States; 4 University of Southern California, Los Angeles, California, United States

## Abstract

Black Americans have experienced multiple health threats during the COVID-19 pandemic, including greater risk of infection compared to Whites. In addition, older adults are more susceptible to worse disease consequences including hospitalization and mortality compared to those who are younger. Racism and economic costs are additional public health crises during this time that have disproportionately impacted Black Americans. Using data from the Uncovering COVID-19 Experiences and Realities (UnCOVER) Study, we examined depressive symptoms in relation to: (1) worry/fear of COVID-19; (2) work loss among household members (being laid off, reduced work hours); and (3) vicarious racism, a particularly salient source of psychosocial stress during the COVID-19 pandemic, including hearing about or seeing acts of racism committed against other race group members. Participants were Black Americans aged 50 years or older (n=300) from five cities (Atlanta, Chicago, Los Angeles, New Orleans, and New York) from May-July 2020. Depression was assessed using the Patient-Reported Outcomes Measurement Information System Short Form. In multivariable linear regression models, all three public health threats were significantly associated with depressive symptoms. When in the model simultaneously, worry/fear of COVID-19 (b=0.30, SE=0.12, p<0.01) and vicarious racism (b=0.62, SE=0.15, p<0.001) showed positive associations; work loss was no longer statistically significant (b=0.62, SE=0.43, p=0.15). When added, the corresponding three-way interaction term was significant (b=0.12, SE=0.04, p<0.01). Synergistic epidemics (“syndemics”) among older Black Americans amplify mental health tolls. Multi-pronged public health strategies are required to address depression in this population.

